# The PPARα/CYP4A14 bile acid pathway is associated with lipid metabolism disorders caused by low birth weight with high-fat diet

**DOI:** 10.29219/fnr.v67.8994

**Published:** 2023-01-24

**Authors:** Fei Zhou, Linquan Yang, Wenwen Sun, Xing Wang, Na Guo, Huijuan Ma, Linlin Yang

**Affiliations:** 1 Department of Internal Medicine, Hebei Medical University, Shijiazhuang, China; 2 Key Laboratory of Metabolic Diseases, Hebei General Hospital, Shijiazhuang, China; 3 Department of Internal Medicine, North China University of Science and Technology, Tangshan, China; 4 Department of Endocrinology, Hebei General Hospital, Shijiazhuang, China

**Keywords:** LBW, lipid metabolism disorders, ACOX2, bile acid, LC-MS/MS

## Abstract

**Purpose:**

To investigate possible mechanisms underlying the greater susceptibility of lipid metabolism disorders in low birth weight (LBW) mice fed with high-fat diets (HFDs).

**Methods:**

LBW mice model was established by using the pregnancy malnutrition method. Male pups were selected from LBW and normal-birth weight (NBW) offspring at random. After 3 weeks of weaning, all offspring mice were fed with HFD. Serum triglycerides (TGs), cholesterol (TC), low density lipoprotein (LDL-C), total bile acid (TAB), non-esterified fatty acid (NEFA), and mice fecal bile acid profiles were measured. Lipid deposition in liver sections was visualized by Oil Red O staining. The weight ratio of liver, muscle, and adiposity was calculated. Tandem mass tag (TMT) combined with LC-MS/MS was used to determine the differentially expressed proteins (DEPs) of liver tissue in two groups. Bioinformatics was used for further analysis of DEPs to screen key target proteins, and then Western Blot (WB) and reverse transcription-quantitative polymerase chain reaction (RT-qPCR) were performed to validate the expressions of DEPs.

**Results:**

LBW mice fed with HFD showed more severe lipid metabolism disorders in the childhood. In contrast to the NBW group, the serum bile acids and fecal ω-muricholic acid (ω-MCA) levels in the LBW group were significantly lower. LC-MS/MS analysis showed that downregulated proteins were associated with lipid metabolism, and further analysis found that these proteins are mainly concentrated in peroxisome proliferation-activated receptor (PPAR) and primary bile acid synthesis signaling pathways and are involved in cellular processes and metabolic processes through binding and catalytic functions. Bioinformatics analysis indicated that the level of Cytochrome P450 Family 46 Subfamily A Member 1 (CYP46A1), PPARα, key factors of cholesterol metabolism and bile acid synthesis, as well as downstream molecules Cytochrome P450 Family 4 Subfamily A Member 14 (CYP4A14), and Acyl-Coenzyme A Oxidase 2 (ACOX2) are markedly different in the liver of LBW individuals fed with HFD, and confirmed by WB and RT-qPCR.

**Conclusion:**

LBW mice are more prone to dyslipidemia probably due to downregulated bile acid metabolism-related PPARα/CYP4A14 pathway, resulting in insufficient metabolism of cholesterol to bile acids, which, in turn, leads to elevated blood cholesterol.

## Popular scientific summary

- LBW with HFD will increase the incidence of lipid metabolism disorders in adulthood.
- LBW mice are more prone to dyslipidemia probably due to downregulated.
- PPARα/CYP4A14 pathway.

There is evidence from early human observational studies that maternal exposure to famine in gestation can increase obesity risk in low birth weight (LBW) offspring as adults (1). LBW offspring may exhibit a period of catch-up growth to compensate for their initial small size, but this catch-up growth may be the major culprit of obesity in adulthood (2, 3). Studies have shown that individuals with LBW who experience catch-up growth periods will have a significantly higher incidence of dyslipidemia, especially high serum cholesterol levels in adulthood (4, 5). Cholesterol is a vital component of cell membranes, essential for the synthesis of steroid hormones, bile acids, and vitamin D (6), but high serum cholesterol is known to increase the risk of metabolic disorders such as diabetes mellitus, hypertension, and cardiovascular diseases (7, 8). The main method by which cholesterol is eliminated from the body is by turning it into bile acid (9). Bile acids are converted from cholesterol through a series of enzyme modifications in the liver (10). Hepatocytes are the only cells that convert cholesterol to bile acids (11). During the elimination of bile acids via the fecal route, new bile acids are formed from hepatic cholesterol (12). Catch-up growth occurs when LBW infants are exposed to higher levels of nutrition postnatally (13), and high-fat diets (HFDs) have been shown to contribute to postnatal catch-up growth. However, the molecular mechanism behind lipid metabolism disorders susceptible phenotype in LBW individuals experienced catch-up growth remains unclear.

In this study, a LBW mouse model was established by using the pregnancy malnutrition method, and HFDs were given to mimic a period of catch-up growth at the same time. Tandem mass tag (TMT) and LC-MS/MS were used to search for differentially expressed proteins (DEPs) in the liver of LBW mice and normal-birth weight (NBW) mice to investigate the possible mechanisms of lipid metabolism disorders caused by LBW fed with HFD, thus establishing a new basis for preventing and treating adult obesity resulted from LBW individuals experienced catch-up growth.

## Materials and methods

### Animals and experimental design

Specific pathogen Free (SPF)-grade adult Institute of Cancer Research (ICR) mice, 20 females and 10 males (6–8 weeks old, 18–22 g), were purchased from Huafukang Biological Technology Co., Ltd. (Beijing, China) and housed in the Animal Barrier System of the Key Laboratory of Metabolic Diseases of Hebei Provincial People’s Hospital. Room temperature was maintained at 20–25°C, relative humidity was at 40–60% with a 12h/12h light/dark cycle. After adaptive feeding for a week with a standard chow diet, female and male mice were housed in the same cage at a ratio of 2:1, and the pregnant mice were identified by the presence of a vaginal plug the following day, which was defined as gestational day 0.5. Pregnant mice were randomly divided into the NBW group and the LBW group at 12.5 days of gestation. From days 12.5 to 18.5 of gestation, the NBW group was given a chow diet, the NBW group was given a chow diet, and the LBW group was given a 50% dietary restriction (the amount of feed offered to the LBW group was half of the NBW group). Within 24 h after delivery, the number of live born and the litter birth weight were recorded. To avoid the influence of female sex hormones on metabolism, only male offspring mice were used for the study. The size of each litter was equalized to seven pups per litter by excluding the heaviest and lightest mice. After 3 weeks of weaning, all offspring mice were fed with HFD. Both the chow diet D12450J and the HFD D12492 were prepared and purchased from Huafukang Biotechnology Co., Ltd. (Beijing, China). Chow diet contains 10% fat, 20% protein, 70% carbohydrate, and 3.85 kcal/g calorific value; HFD includes 45% fat, 20% protein, 35% carbohydrate, and 5.24 kcal/g calorific value. Animal experimental procedures were approved by the Animal Ethics Committee of Hebei Provincial People’s Hospital.

### Sample collection and preparation

After 17 weeks of high-fat intervention, the mice fasted for 12 h were intraperitoneally anesthetized with 1% sodium pentobarbital (60 mg/kg). Blood samples were collected from the orbital venous plexus and centrifuged at 3,000 rpm for 10 min at 4°C, and then the supernatant was collected and preserved at –80°C refrigerator (Qingdao Haier Biomedical Co., Ltd, Qingdao, China) for standby application. The liver, muscle, and white fat of each mouse were taken out and weighed, rapidly frozen in liquid N2 and then move to the −80°C refrigerator for subsequent experiments. Fecal samples were collected and stored in a −80°C refrigerator until bile acid profile measurement.

### Detection of serum indicators

Serum triglycerides (TGs), cholesterol (TC), low-density lipoprotein (LDL-C), non-esterified fatty acid (NEFA), and total bile acids (TAB) were measured by the corresponding assay kit (Nanjing Jiancheng Bioengineering Institute, Nanjing, China), as described by the manufacturer.

### Oil Red O staining of liver tissue

Lipid deposition in frozen liver sections was visualized by Oil Red O staining. Light microscopy was used to observe and photograph the stained sections.

### Detection of mice fecal bile acid profiles

The detection of mice fecal bile acid profiles was performed by Shanghai Zhongke New Life Biotechnology Co., Ltd.

### Sample preparation

The sample was grinded by liquid nitrogen into powder, and four volumes of lysis buffer (8 M urea, 1% Protease Inhibitor Cocktail) were added to the powder, followed by sonication three times on ice using a high intensity ultrasonic processor (Scientz, Ningbo, China). After centrifugation at 12,000 g at 4°C for 10 min, the supernatant was collected, and the protein concentration was determined using a bicinchoninic acid (BCA) kit, according to the manufacturer’s instructions. For digestion, the protein solution was reduced with 5 mM dithiothreitol for 30 min at 56°C and alkylated with 11 mM iodoacetamide for 15 min at room temperature in darkness. The protein sample was then diluted by adding 100 mM tetraethylammoniumbromide (TEAB) to urea concentration less than 2M. Finally, trypsin was added at a 1:50 trypsin-to-protein mass ratio for the first digestion overnight and a 1:100 trypsin-to-protein mass ratio for a second 4 h-digestion. After trypsin digestion, the peptide was desalted by Strata X C18 SPE column (Phenomenex, Torrance, California, USA) and vacuum-dried. The peptide was reconstituted in 0.5 M TEAB and processed according to the manufacturer’s protocol for the TMT kit/iTRAQ kit.

### LC-MS/MS analysis

The tryptic peptides were dissolved in 0.1% formic acid (solvent A; Fluka, St. Louis, USA) and directly loaded onto a home-made reversed-phase analytical column (15-cm length, 75 μm i.d.). The gradient was comprised of an increase from 6 to 23% solvent B (0.1% formic acid in 98% acetonitrile) over 26 min, 23 to 35% in 8 min, and climbing to 80% in 3 min then holding at 80% for the last 3 min, all at a constant flow rate of 400 nL/min on an EASY-nLC 1,000 ultra performance liquid chromatography (UPLC) system.

The peptides were subjected to nanospray ionization (NSI) source followed by tandem mass spectrometry (MS/MS) in Q ExactiveTM Plus (Thermo Fisher Scientific, San Jose, CA, USA) coupled online to the UPLC. The electrospray voltage applied was 2.0 kV. The m/z scan range was 350 to 1,800 for full scan, and intact peptides were detected in the Orbitrap at a resolution of 70,000. Peptides were then selected for MS/MS using Normalized Collision Energy (NCE) setting as 28, and the fragments were detected in the Orbitrap at a resolution of 17,500. A data-dependent procedure alternated between one MS scan followed by 20 MS/MS scans with 15.0s dynamic exclusion. Automatic gain control (AGC) was set at 5E4. The fixed first mass was set as 100 m/z.

### Bioinformatic analysis

The Gene Ontology (GO) was used to provide protein annotation and classification, which was derived from the UniProt-GOA database (http://www.ebi.ac.uk/GOA/). If some identified proteins were not annotated by the UniProt-GOA database, the InterProScan soft would be used to annotated protein’s GO functional based on the protein sequence alignment method. Kyoto Encyclopedia of Genes and Genomes (KEGG) database was used to annotate protein pathways. Subcellular localization prediction was performed by using Wolfpsort soft.

### Reverse transcription-quantitative polymerase chain reaction

The total RNA of liver tissues was extracted by using TRIzol (DP424, Tiangen Biotech Co., Ltd., Beijing, China), according to the manufacturer’s protocol. A Nanodrop ND-1000 spectrophotometer (Thermo Fisher Scientific, USA) was used to measure RNA quality and quantity. The total RNA was reverse-transcripted to cDNA using the First Strand cDNA Synthesis kit (KR116, Tiangen Biotech Co., Ltd., Beijing, China). Expression levels detection of each gene was performed on Applied Biosystems 7500 Real-Time PCR System (Applied Biosystems, Foster City, CA, USA) using a Super Real PreMix Plus (SYBR Green) fluorescence quantitative premixing kit (FP205, Tiangen Biotech Co., Ltd., Beijing, China) through the 2-step method. The 2-ΔΔCt method was used to calculate and normalize relative gene expression. The standard cycling conditions were as follows: Pre-denaturing at 95°C for 15 min, followed by 40 cycles at 95°C for 10 s and 60°C for 32 s. Primers were designed and synthesized by Tiangen Biotech Co., Ltd. (Beijing, China), and the sequences of these primers are shown in Table 1.

**Table 1:** Primer sequences used for gene expression analysis

Gene	Primer sequence (5ʹ-3ʹ)
GAPDH	F: CCTCGTCCCGTAGACAAAATG R: TGAGGTCAATGAAGGGGTCGT
PPARα	F: CACTACGGAGTTCACGCATGT R: GTGACATCCCGACAGACAGGC
CYP4A14	F: GAAGAAGTGGTTCCAGCATCG R: CAGGCGAAAGAAAGTCAGGTTG
ACOX2	F: GTCATACACCTTCAGGCTGCTAA R: AGGCATAGAGGTCACAGAGGTTCT
CYP46A1	F: TGGAGGAGGAGACCTTGATTGA R: GGAAAGTAAGTGAACCGTGGCT

PPARα, peroxisome proliferation-activated receptor alpha; ACOX2, Acyl-Coenzyme A Oxidase 2; CYP4A14, Cytochrome P450 Family 4 Subfamily A Member 14; CYP46A1, Cytochrome P450 Family 46 Subfamily A Member 1.

### Western blot assay

Total protein was extracted from liver tissue using high-efficiency radio immunoprecipitation assay (RIPA) lysis (Solarbio, Beijing, China). Protein concentrations were quantified using the BCA Protein Assay Kit (Solarbio, Beijing, China). The samples were separated using sodium dodecyl sulfate polyacrylamide gel electrophoresis (SDS-PAGE) and then transfer to polyvinylidene fluoride (PVDF) membranes (Merck Millipore, Billerica, MA, USA). Then, the membranes were blocked using 5% skimmed milk powder (Solarbio, Beijing, China) at room temperature for 2 h. Primary antibodies were incubated overnight at 4°C. The antibody dilution ratios are as follows: glyceraldehyde-3-phosphate dehydrogenase (GAPDH) (10494-1-AP; anti-rabbit; 1: 10,000; ProteinTech, Wuhan, China), peroxisome proliferation-activated receptor alpha (PPARα) (15540-1-AP; anti-rabbit; 1: 1,000; ProteinTech, Wuhan, China), Cytochrome P450 Family 4 Subfamily A Member 14 (CYP4A14) (ab3573; anti-rabbit; 1: 1,000; Abcam, USA), Acyl-Coenzyme A Oxidase 2 (ACOX2) (ab197808; anti-rabbit; 1: 1,000; Abcam, USA), and Cytochrome P450 Family 46 Subfamily A Member 1 (CYP46A1) (12486-1-AP; anti-rabbit; 1: 1,000; ProteinTech, Wuhan, China). Subsequent to washing, the membranes were incubated with horseradish peroxidase-conjugated goat anti-rabbit IgG (511203; 1: 5,000; Zhengneng Biotechnology, Chengdu, China). The Enhanced Chemiluminescence (ECL) supersensitive substrate chemiluminescence detection kit was then used to perform blots detection, and the Image J software was used to calculate the gray value of protein bands.

### Statistical analysis

Data analysis was conducted using SPSS statistics 26.0. A Student’s t test was conducted for normal distribution and equal variance data, and non-parametric tests were used for data that did not satisfy normality or equivariance criteria. *P* < 0.05 was considered a statistically significant difference. The GraphPad Prism 8.0.1 software (San Diego, CA, USA) was used for statistical mapping.

## Results

### Establishment of LBW animal model

**Fig. 1:**
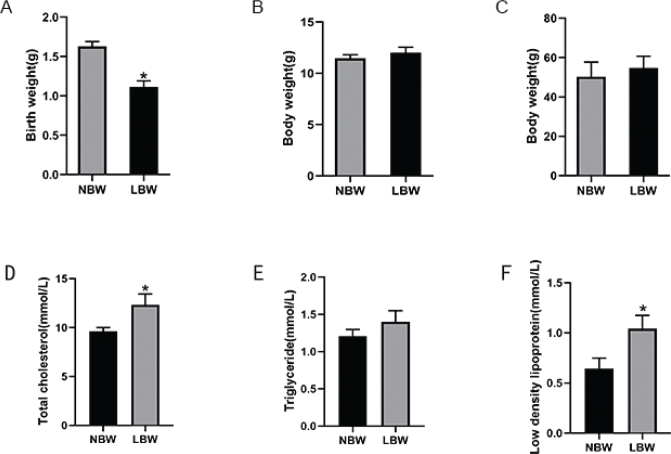
(A) Birth weight, (B) body weight at age 3 weeks, (C) body weight at age 20 weeks, (D) TC, (E) TG, and (F) LDL-C of mice in two groups. *P < 0.05 vs NBW (Student’s t test). TG, triglycerides; TC, cholesterol; LDL, low-density lipoprotein; NBW, normal-birth weight.

As shown in Figure 1A, the birth weight of mice in the LBW group (1.1143 ± 0.20 g) was prominently lower than that in the NBW group (1.6286 ± 0.16 g), indicating the LBW mice model was successfully established by the pregnancy malnutrition method. The body weight of mice in the LBW group caught up with the NBW group at age 3 weeks, and the body weight of mice in the two groups was not obviously different after 17 weeks of HFD intervention (age 20 weeks) (Figure 1B, C).

### LBW mouse model developed lipid metabolism disorders

**Fig. 2:**
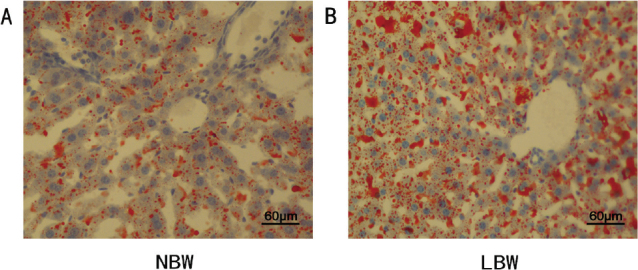
Oil Red O staining of liver tissue in two groups.

The levels of TC and LDL-C of the LBW group were significantly higher than those in the NBW group after 17W high-fat intervention (Figure 1D, F). The TG (Figure 1E) and NEFA (Figure 6D) levels of NBW and LBW groups showed no significant differences. According to the Oil Red O staining of the liver, the red lipid droplets in hepatocytes of the LBW group (Figure 2B) were more than the NBW group (Figure 2A), indicating more severe lipid metabolism disorders in mice of the LBW group.

### The identification of DEPs

**Fig. 3:**
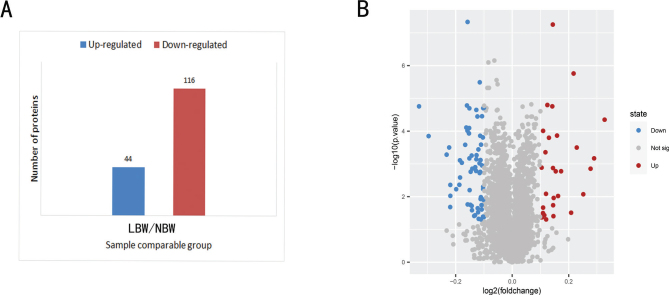
Quantitative information on differential protein identification; (A) histogram of the number distribution of DEPs; (B) volcano plot of DEPs. DEPs, differentially expressed proteins.

*P* value < 0.05 and fold change >1.2 were set as the threshold for significant upregulation in expression, and *P* value < 0.05 and fold change < 1/1.2 were set as the threshold for significant downregulation in expression. A total of 160 DEPs were obtained from NBW and LBW groups (44 were upregulated and 116 were downregulated), as displayed in Figure 3A. In order to demonstrate the DEPs intuitively, the R Language ggplots2 software package was used for plotting the volcano plot (Figure 3B).

### GO annotation and subcellular structure localization of DEPs

**Fig. 4:**
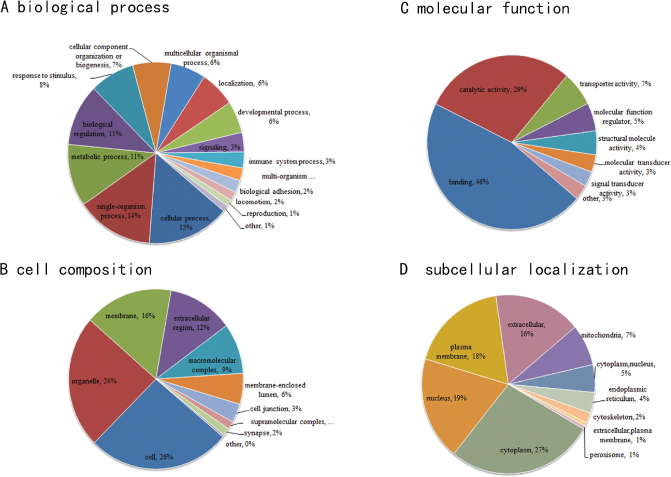
(A) The enrichment analysis results of BP; (B) the enrichment analysis results of CC; (C) the enrichment analysis results of MF; (D) subcellular localization chart of DEPs. BP, biological process; CC, cell composition; MF, molecular function; DEPs, differentially expressed proteins.

The GO annotation includes biological process (BP), cell composition (CC), and molecular function (MF), which aids in identifying functions of DEPs. BP results showed that the DEPs of the two groups were mainly involved in cellular process, single-organism process, metabolic process, and biological regulation (Figure 4A). The CC of the DEPs mainly focused on cell, organelle, and membrane (Figure 4B). In the MF analysis, these proteins are mainly involved in binding, catalytic activity, transporter activity, and MF regulator (Figure 4C). As shown in the pie chart of Figure 4D, the subcellular structure localization distribution of the two groups of DEPs mainly distributed in the cytoplasm, nucleus, plasma membrane, extracellular, mitochondria, cytoplasm, and so on.

### Protein domain analysis

**Fig. 5:**
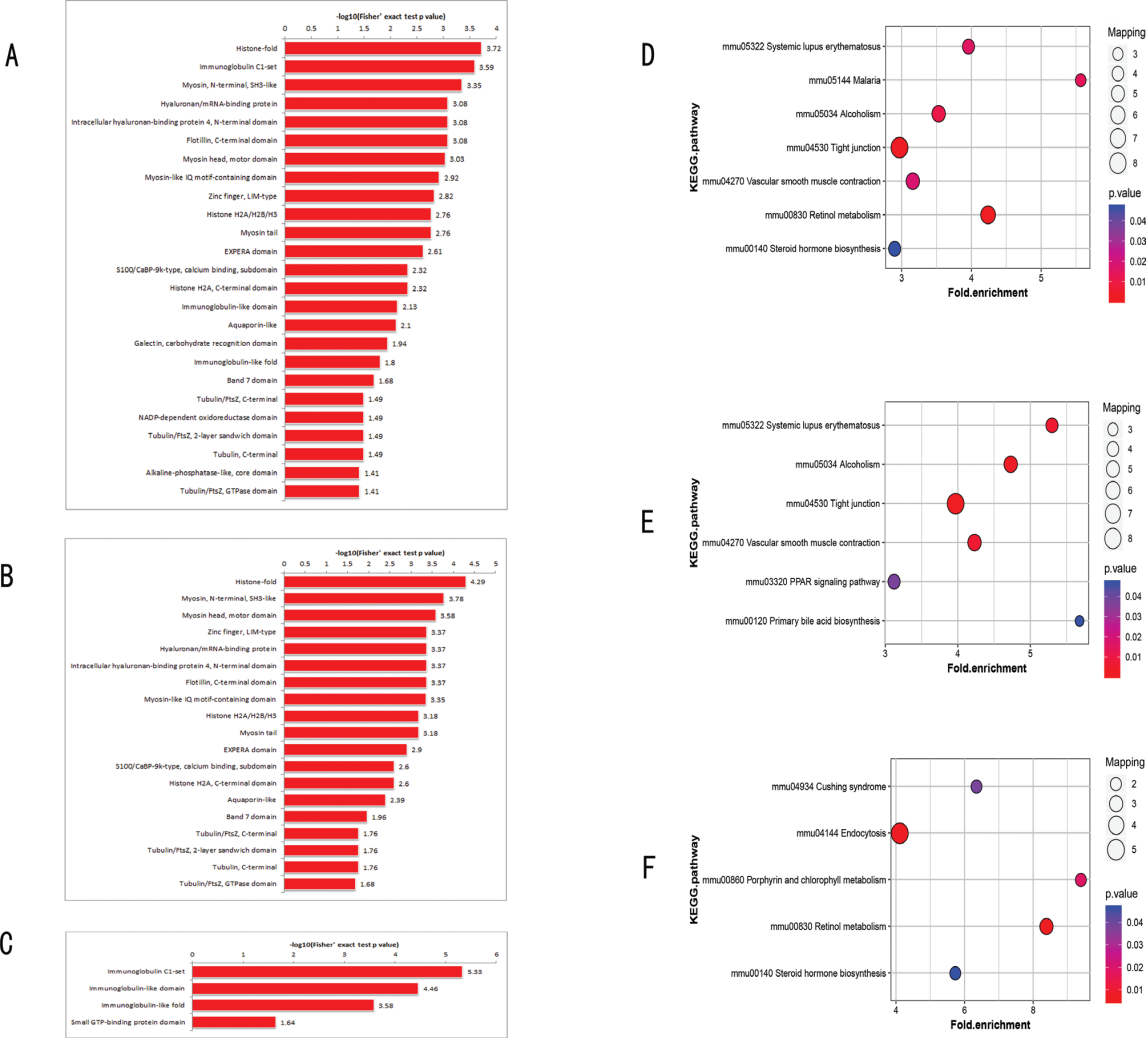
(A) The domain of all DEPs; (B) the domain of downregulated DEPs; (C) the domain of upregulated DEPs; (D) the KEGG pathway enrichment of all DEPs; (E) the KEGG pathway enrichment of downregulated DEPs; (F) the KEGG pathway enrichment of upregulated DEPs. DEPs, differentially expressed proteins; KEGG, Kyoto Encyclopedia of Genes and Genomes.

According to the protein domain analysis results, the domains of DEPs in the NBW and LBW group were predominantly concentrated in histone-fold; immunoglobulin C1-set; myosin, N-terminal, and SH3-like; hyaluronan/ messenger RNA (mRNA)-binding protein; intracellular hyaluronan-binding protein 4 and N-terminal domain; flotillin and C-terminal domain; myosin head, motor domain; and so on (Figure 5A). The downregulated DEPs were focused on histone-fold; myosin, N-terminal, and SH3-like; myosin head and motor domain; zinc finger, lineage 11 (Lin-11), insulin I (Isl-1) and mechanotransduction 3 (Mec-3) (LIM)-type, and hyaluronan/mRNA-binding protein (Figure 5B). The upregulated DEPs were focused on immunoglobulin C1-set; immunoglobulin-like domain; immunoglobulin-like fold and small guanosine triphosphate (GTP)-binding protein domain (Figure 5C).

### KEGG signaling pathway enrichment analysis

KEGG pathway analysis results showed that the DEPs in LBW and NBW groups were involved in retinol metabolism, tight junction, alcoholism, malaria, systemic lupus erythematosus, vascular smooth muscle contraction, and steroid hormone biosynthesis (Figure 5D). The downregulated DEPs were principally involved in tight junction, alcoholism, vascular smooth muscle contraction, systemic lupus erythematosus, PPAR signaling pathway, and primary bile acid biosynthesis (Figure 5E). The upregulated DEPs were mainly involved in retinol metabolism, endocytosis, porphyrin and chlorophyll metabolism, Cushing syndrome, and steroid hormone biosynthesis (Figure 5F).

### The weight ratio of organs, serum TBA levels, and fecal bile acid spectrum in two groups of mice

**Fig. 6:**
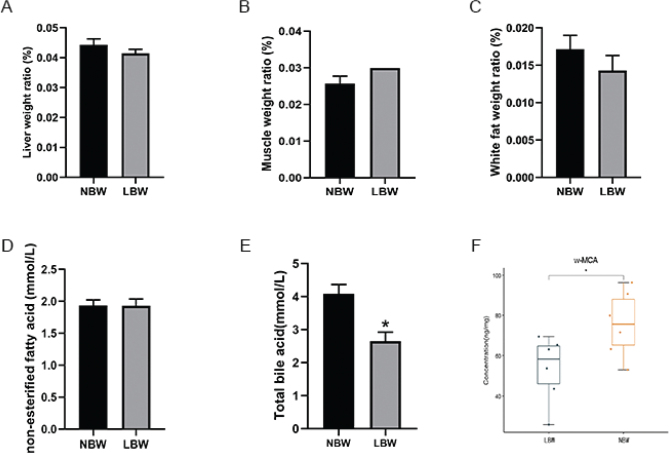
The weight ratio of (A) liver, (B) muscle, and (C) white fat of mice in two groups; (D) comparison of serum NEFA of mice in two groups; (E) comparison of serum TBA of mice in two groups; *P < 0.05 vs NBW (Student’s t test). (F) Comparison of fecal bile acid spectrum in two groups. NEFA, non-esterified fatty acid; NBW, normal-birth weight.

The weight ratio of liver, muscle, and white fat did not differ between two groups of mice (Figure 6A–C). The serum TBA levels of mice in the LBW group were significantly lower than those in the NBW group after 17W HFD intervention (Figure 6E). ω-Muricholic Acid (ω-MCA) in the fecal of the LBW group was also clearly lower than that of the NBW group (Figure 6F).

### Verification of the PPARα/CYP4A14 bile acid signaling pathway

**Fig. 7:**
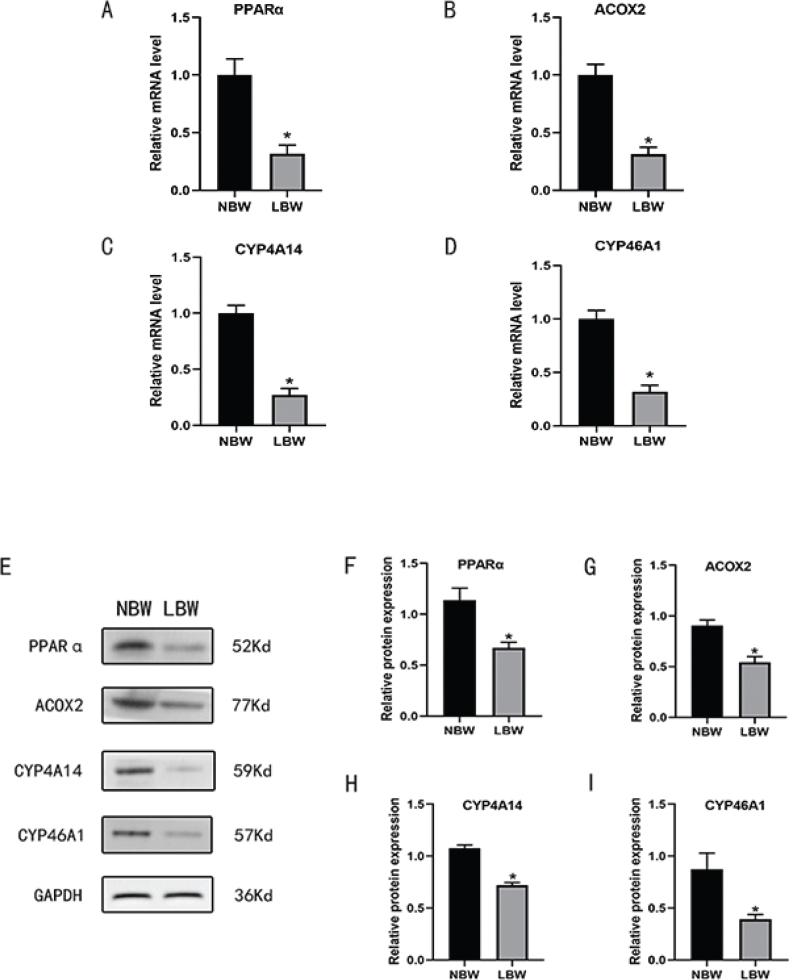
(A) The mRNA expression of PPARα; (B) the mRNA expression of ACOX2; (C) the mRNA expression of CYP4A14; (D) the mRNA expression of CYP46A1. *P < 0.05 vs NBW; (E) the protein bands of PPARα, ACOX2, CYP4A14, CYP46A1, and GAPDH; (F) the relative protein expression of PPARα; (G) the relative protein expression of ACOX2; (H) the relative protein expression of CYP4A14; (I) the relative protein expression of CYP46A1. *P < 0.05 vs NBW (Student’s t test). PPARα, peroxisome proliferation-activated receptor alpha; ACOX2, Acyl-Coenzyme A Oxidase 2; CYP4A14, Cytochrome P450 Family 4 Subfamily A Member 14; CYP46A1, Cytochrome P450 Family 46 Subfamily A Member 1; NBW, normal-birth weight.

Reverse transcription-quantitative polymerase chain reaction (RT-qPCR) results revealed that mRNA expression of PPARα, ACOX2, CYP4A14, and CYP46A1 decreased in the LBW group. The trends of PPARα, ACOX2, CYP4A14, and CYP46A1 protein expressions were in accordance with those detected by RT-qPCR (Figure 7).

## Discussion

LBW infants exposed to HFD showed a period of catch-up growths after birth, which was considered to be a strong predictor for later developments of metabolic diseases such as obesity in later life (14–17). To unravel the molecular mechanisms of it, we established the LBW model using the pregnancy malnutrition method. According to our results, the birth weights of mice in the LBW group were significantly lower than those in the NBW group (Figure 1A), indicating that the LBW animal model was set up successfully. The mice in the LBW group exhibited significant catch-up growth, and the body weight of mice in the LBW group did not differ from that in the NBW group at age 3 weeks (Figure 1B). After 17 weeks of HFD, the body weight was not significantly different between the two groups (Figure 1C).

Several clinic studies suggested that LBW individuals on HFD showed an increased trend toward serum TC and liver fat compared to NBW on HFD (18–20). Animal studies also showed that LBW is a risk factor for increased hepatic cholesterol (21). These were in accordance with our results. After 17W of high-fat intervention, the levels of TC and LDL-C in the LBW group were markedly higher than those in the NBW group (Figure 1D, F). Oil Red O staining results also demonstrated that the red lipid droplets in hepatocytes of the LBW group were more than that of the NBW group, indicating more severe hepatic steatosis (Figure 2).

In this study, TMT combined with LC-MS/MS was used to explore the underlying molecular mechanism that LBW mice fed with HFD were more prone to develop lipid metabolism disorders. A total of 160 DEPs were obtained, including 44 upregulated proteins and 116 downregulated proteins (Figure 3). According to the GO analysis results, the DEPs of the two groups were involved in metabolic processes and biological regulation (Figure 4A–C). KEGG pathway analysis results revealed that the downregulated DEPs focused on primary bile acid biosynthesis and PPAR signaling pathway (Figure 5D–F). Primary bile acids are synthesized directly from cholesterol in the liver by the hepatocytes, and secondary bile acids are derived by bacteria in the caecum and colon by modifications of the primary bile acids (22). In mice, primary BAs were conjugated by glycine and taurine and dehydroxylated to form ω-MCA as a secondary BA (23). Our results showed that after 17W of high-fat intervention, the serum TBA level in the LBW group was clearly lower than that in the NBW group (Figure 6E). Fecal ω-MCA of the LBW group was distinctly lower than that of the NBW group (Figure 6F). We speculated that the reduction of bile acids biosynthesis could potentially underlie the elevation of cholesterol. This was in line with the findings of Berger’s group. They identified that liver cholesterol content was elevated in the liver not by increased cholesterol synthesis, but by a decrease in bile acids and fecal cholesterol excretion (24).

Based on the results of the bioinformatics analysis, we found that CYP46A1, PPARα, and their target molecules CYP4A14 and ACOX2, which are key factors of cholesterol metabolism and bile acid biosynthesis, were significantly different in LBW individuals. Cyp46a1 encodes a member of the cytochrome P450 superfamily of enzymes (25), which is the major regulator of cholesterol elimination (26). Studies showed that downregulation of the expression of Cyp46A1 increased the concentration of cholesterol (27). PPARα, highly expressed in the liver (28), is reported to be a crucial transcription factor that regulates lipid metabolism and influences bile acid biosynthesis (29–31). The activation of PPARα has benefits in improving systemic lipid metabolism (32, 33). Specifically, PPARα serves a role in the clearance of circulating cholesterol via upregulating CYP expression in hepatocytes (34). On the contrary, blocking the PPARα signaling pathway could induce lipid accumulation in hepatocytes. On the contrary, blocking the PPARα signaling pathway could induce lipid accumulation in hepatocytes (35). Therefore, the reduction of PPARα may lead to impaired clearance of cholesterol, leading to the initiation and development of hyperlipidemia. CYP4A14 is also highly expressed in liver (36) and serves as a classic target gene of PPARα (37), so PPARα is likely responsible for the constitutive expression of CYP4A14 in the liver (38). ACOX2 is a member of the ACOX protein family (39), which is the rate-limiting enzyme in the synthesis of bile acid precursor molecules (40). ACOX2 produces C24 bile acids by shortening C27 cholesterol derivatives, thereby catalyzing the decomposition of cholesterol into bile acids in the liver (41). By using RT-qPCR and Western Blot (WB) technology, we confirmed that the protein and mRNA expression levels of PPARα, CYP4A14, and ACOX2 in the liver of LBW mice with HFD were reduced compared with the NBW mice fed with HFD (Figure 7). Combined with our bioinformatics analysis results, it can be inferred that the downregulated bile acid metabolism-related PPARα/CYP4A14 pathway in the liver may lead to the incidence of lipid metabolism disorder in LBW infants fed with HFD as adults.

## Conclusion

Here, we revealed, by proteomics techniques combined with bioinformatics analysis, LBW mice fed with HFD are more prone to dyslipidemia as adults probably due to the downregulated bile acid metabolism-related PPARα/CYP4A14 pathway, resulting in insufficient metabolism of cholesterol to bile acids, which, in turn, leads to elevated blood cholesterol. This finding provided a significant clue to reveal the underlying mechanism of the formation of lipid metabolism disorders in LBW individuals during adulthood.

## Data Availability

The analyzed datasets generated about animal experiment during the study are available from the corresponding author on reasonable request.
